# *TMPRSS2/ERG* Promotes Epithelial to Mesenchymal Transition through the *ZEB1*/*ZEB2* Axis in a Prostate Cancer Model

**DOI:** 10.1371/journal.pone.0021650

**Published:** 2011-07-01

**Authors:** Orit Leshem, Shalom Madar, Ira Kogan-Sakin, Iris Kamer, Ido Goldstein, Ran Brosh, Yehudit Cohen, Jasmine Jacob-Hirsch, Marcelo Ehrlich, Shmuel Ben-Sasson, Naomi Goldfinger, Ron Loewenthal, Ephraim Gazit, Varda Rotter, Raanan Berger

**Affiliations:** 1 Sheba Cancer Research Center, Sheba Medical Center, Tel Hashomer, Ramat Gan, Israel; 2 Department of Developmental Biology and Cancer Research, Institute for Medical Research Israel-Canada (IMRIC), The Hebrew University-Hadassah Medical School, Jerusalem, Israel; 3 Department of Molecular Cell Biology, Weizmann Institute of Science, Rehovot, Israel; 4 Department of Cell Research and Immunology, George S. Wise Faculty of Life Sciences, Tel Aviv University, Tel Aviv, Israel; 5 Tissue Typing Unit, Sheba Medical Center, Tel Hashomer, Ramat Gan, Israel; Roswell Park Cancer Institute, United States of America

## Abstract

Prostate cancer is the most common non-dermatologic malignancy in men in the Western world. Recently, a frequent chromosomal aberration fusing androgen regulated *TMPRSS2* promoter and the *ERG* gene (*TMPRSS2/ERG*) was discovered in prostate cancer. Several studies demonstrated cooperation between *TMPRSS2/ERG* and other defective pathways in cancer progression. However, the unveiling of more specific pathways in which *TMPRSS2/ERG* takes part, requires further investigation. Using immortalized prostate epithelial cells we were able to show that *TMPRSS2/ERG* over-expressing cells undergo an Epithelial to Mesenchymal Transition (EMT), manifested by acquisition of mesenchymal morphology and markers as well as migration and invasion capabilities. These findings were corroborated *in vivo*, where the control cells gave rise to discrete nodules while the *TMPRSS2/ERG*-expressing cells formed malignant tumors, which expressed EMT markers. To further investigate the general transcription scheme induced by *TMPRSS2/ERG*, cells were subjected to a microarray analysis that revealed a distinct EMT expression program, including up-regulation of the EMT facilitators, *ZEB1* and *ZEB2,* and down-regulation of the epithelial marker *CDH1*(E-Cadherin). A chromatin immunoprecipitation assay revealed direct binding of *TMPRSS2/ERG* to the promoter of *ZEB1* but not *ZEB2*. However, *TMPRSS2/ERG* was able to bind the promoters of the *ZEB2* modulators, *IL1R2* and *SPINT1*. This set of experiments further illuminates the mechanism by which the *TMPRSS2/ERG* fusion affects prostate cancer progression and might assist in targeting *TMPRSS2/ERG* and its downstream targets in future drug design efforts.

## Introduction

Prostate cancer is one of the most frequent cancers in Men. Close to 30,000 patients are expected to die from the disease in the USA each year. A major advance in this research field is a recent discovery that frequent over-expression of E Twenty Six (ETS)-related proto-oncogenes may be driven by androgen receptor as a consequence of common genomic rearrangements. The predominant form of the aforementioned fusions with a frequency of ∼85% [1], is the fusion between exon 1 from TMPRSS2 and exons 4–9 from the *ERG* gene, which occurs either by a deletion of 3 mega bases region separating these genes [2], or via an interchromosomal translocation [3], [4]. As this fusion is already evident in Prostatic Intraepithelial Neoplasia (PIN) [5], investigating this fusion may hold the key towards understanding the mechanisms involved in early phases of prostate cancer.

Since its discovery [6], the *TMPRSS2/ERG* fusion has been extensively studied in several aspects, including early diagnosis, prognosis, contribution to cancer progression and even as a target for cancer therapy [7]. According to long term clinical studies performed on a large cohort of patients, it seems that *TMPRSS2/ERG* expression is associated with a more aggressive form of prostate cancer [8], [9]. Further studies have shown a role for *TMPRSS2/ERG* fusion in tumorigenesis in terms of proliferation, invasion and cancer initiation and progression [10], [11], [12], [13]. In general, it appears that cell proliferation is not necessarily promoted via *TMPRSS2/ERG* expression. As for tumorigenesis, the data is inconclusive. While knocking-down endogenous *TMPRSS2/ERG* in the VCaP prostate-derived cancer cells resulted in a reduction of both tumor uptake and volume [13], [14], transgenic mice harboring *TMPRSS2/ERG* in their genome either developed PIN [10], [15] or reveal no histological evidence of PIN or invasive cancer [11], [16]; depending on the specific model used in the study and the interpretation of the data. Despite the disagreement concerning the role of *TMPRSS2/ERG* in cancer initiation, cell invasion was suggested to be a consequence of *TMPRSS2/ERG* fusion both *in vitro* and *in vivo* [10], [13], [15]. Interestingly, an *in silico* study revealed that *TMPRSS2/ERG* co-expressed with histone deacetylase 1 (HDAC1) is coupled with down regulation of its known target [17]. This finding implies that *TMPRSS2/ERG* is associated with epigenetic reprogramming. Accordingly, in a follow-up study performed by the same group, HDACi, and HDAC specific inhibitors, compromised TMPRSS2*/ERG* expression or activity in ERG positive cells, *in vitro* [17], [18]. In addition, recent findings demonstrated a cooperation between TMPRSS2/ERG fusion and deregulated activity of cancer-related pathways, such as PTEN [19], PI3-Kinase [16], and AKT or AR [20]. More recently, TMPRSS2/ERG was shown to mediate Epithelial to Mesenchymal Transition (EMT) through the induction of WNT signaling components [21]. Taken together, it could be surmised that other *TMPRSS2/ERG*-mediated pathways, might be converged at the same endpoint, namely, EMT and invasion; and therefore discovering new pathways through which *TMPRSS2/ERG* exert this effect is of great importance. The main motivation of this study is therefore to unravel such *TMPRSS2/ERG* related pathways in the context of prostate cancer. In a previous work we established immortalized and tumorigenic human prostate epithelial cells (PrECs) lines of defined genetic constitution [22]. Similarly, in the presented study, we generated genetically modified PrECs to serve as a background on which the effects of the *TMPRSS2/ERG* fusion could be genuinely studied. We found that TMPRSS2/ERG executes a distinct EMT expression program which is mainly governed by a direct activation of *ZEB1* and an indirect induction of *ZEB2* through *SPINT1* and *IL1R2* modulation, leading to an EMT phenotype *in vitro* and *in vivo*.

## Results

### Establishment of immortalized PrECs cultures

**Figure 1:**
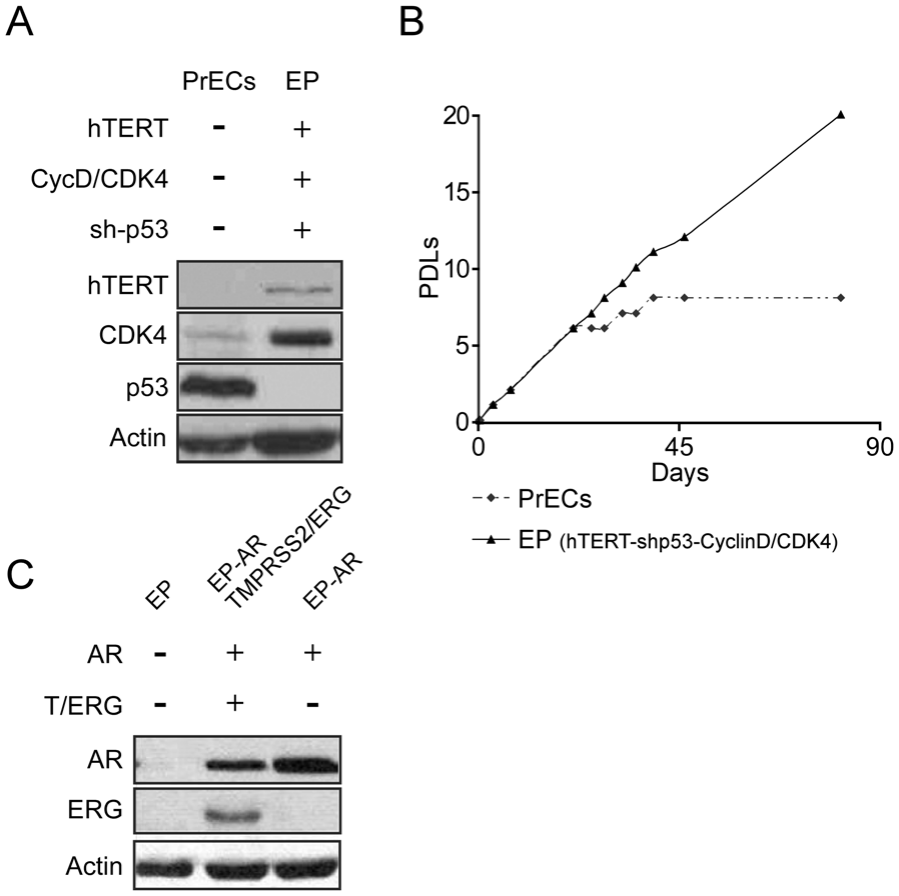
Prostate epithelial cells immortalization. To induce immortalization, cells were introduced with the telomerase catalytic subunit hTERT, and both the p53 and pRB pathways were perturbed using p53 knockdown and over-expression of cyclinD/CDK4, respectively. (B) Primary PrECs, as well as hTERT/shp53/CyclinD-CDK4 – overexpressing cells (EP cells), were sequentially passaged and counted. Population Doublings (PDLs) were calculated using the formula: PDLs = log(cell output/cellinput)/log2. (C) EP cells were introduced with AR and either *TMPRSS2/ERG* (EP-AR TMPRSS2/ERG) or an empty vector (EP-AR). AR and ERG protein levels were measured by Western blot. Actin was used as a loading control.

In order to investigate the impact of *TMPRSS2/ERG* in a genetically modified environment we sought to establish an immortalized PrECs culture. Normal prostate epithelial cells were produced from a human prostatectomy specimen and were subsequently grown in culture. To induce immortalization, cells were introduced with the telomerase catalytic subunit hTERT, and both the p53 and pRB pathways were perturbed by p53 knockdown and over-expression of CyclinD/CDK4 chimera, respectively, giving rise to an immortal cell line designated as EP (Figure 1A and B). Next, the immortalized cells were infected with retroviruses encoding either *TMPRSS2/ERG* or empty-vector control (Figure 1C). ERG protein level was comparable with its previously reported expression level in cell lines and cancer samples [23], [24]. Notably, *TMPRSS2/ERG* alone or in combination with hTERT and/or p53 knockdown was not sufficient to induce immortalization (data not shown). Finally, following a previous report that the combination of Androgen Receptor (AR) and high levels of ERG promotes the development of a more poorly differentiated, invasive adenocarcinoma than either gene alone [20]; AR was introduced into the *TMPRSS2/ERG*-expressing cells as well as into their empty-vector controls (Figure 1C).

### A role for *TMPRSS2/ERG* in epithelial to mesenchymal transition *in vitro*

**Figure 2:**
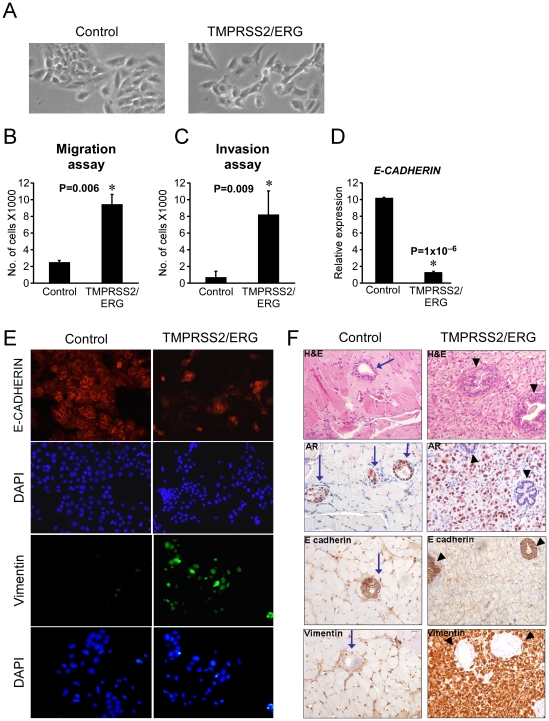
*TMPRSS2/ERG* promotes EMT in prostate epithelial cells. (A) For morphological comparison, cells were photographed using a light microscope. (B) Cells were seeded in transwells and their migratory capacity towards FCS was measured by counting the migrating cells. (C) The same setup as in (B) was used with matrigel-coated transwells in order to compare cell invasiveness. (D) Cells were analyzed for CDH1 (E-Cadhein) expression using QRT-PCR. The results are presented as mean ±SD of a triplicate from a representative experiment. * denotes a significant differential expression of the gene compared to the control. (E) Cells were plated on slides and stained for CDH1 and Vimentin. DAPI was used to visualize nuclei. (F) Cells were implanted into murine prostate glands. Glands were removed 68 days after implantation, sectioned and either stained with Hematoxilin and Eosin (H&E) or with antibodies against human AR, CDH1 and Vimentin. (X400 Magnifications). Note that EP-AR (Control) cells formed discrete prostate nodules (Blue arrows), which are positively stained with the human specific anti-AR antibody (α-hAR). In contrast, EP-AR TMPRSS2/ERG-derived tumors, which are positively stained with α-hAR antibody, engulfed the α-hAR-negative murine nodules (Black arrowheads).

Comparing the morphology of EP-AR and EP-AR *TMPRSS2/ERG* cell lines under a light microscope, we observed that EP-AR *TMPRSS2/ERG* cells acquired fibroblastic-like characteristics, as they demonstrated a more elongated morphology and a scattered density compared to their isogenic controls, which exhibited higher degree of adherence between neighboring cells (Figure 2A). The observed alterations, which are characteristic features of EMT [25], [26], coupled with previous reports associating *TMPRSS2/ERG* with EMT and invasion [10], [21], prompted us to examine whether in addition to the morphological changes, cells were also granted with motility and invasion capacities. To this end, cells were seeded in transwells with serum-free media and their migration towards serum-supplemented media was assessed. As shown in Figure 2B, *TMPRSS2/ERG*-expressing cells exhibited an enhanced migratory capacity. The same experiment was repeated using matrigel-coated wells in order to examine the cells ability to penetrate and invade a dense surface. Once again, invasion ability was significantly more discernible in the *TMPRSS2/ERG* expressing cells (Figure 2C). The loss of *CDH1* (E-Cadherin) is considered to be the most fundamental event during EMT [27]. We therefore measured the levels of *CDH1* mRNA and protein using QRT-PCR and immunofluorescence staining, respectively. Indeed, EP-AR *TMPRSS2/ERG* cells demonstrated a marked reduction in the levels of *CDH1* mRNA (Figure 2D) and protein (Figure 2E). Additionally, *VIM* (Vimentin), a known mesenchymal marker was found to be elevated in the *TMPRSS2/ERG*-expressing cells (Figure 2E). In sum, our data suggest that *TMPRSS2/ERG* overexpression provokes an epithelial to mesenchymal transition *in vitro*.

### The effect of *TMPRSS2/ERG* on tumorigenesis

In an attempt to extend the previous observation to an *in vivo* model; we either injected the genetically-modified cell lines subcutaneously or implanted them orthotopically into the prostate of nude mice. Sixty eight days following the implantation, tumors were removed, sectioned and stained for EMT markers. Comparing the orthotopic implantation sites of the distinct cell lines revealed that hTERT/shp53/CDK4-immortalized PrECs (EP cells) did not form tumors (Data not shown), while EP-AR formed discrete nodules interspersed throughout the murine prostate (Figure 2F, indicated by blue arrows). Notably, EP-AR *TMPRSS2/ERG* cells formed large malignant tumors, which surrounded the normal murine prostate nodules (Figure 2F, black arrowheads). Moreover, EP-AR-derived nodules demonstrated positive staining for the epithelial marker CDH1, and failed to stain for the mesenchymal marker VIM (Figure 2F, blue arrows). A mirror image was evident in EP-AR *TMPRSS2/ERG*-derived tumors, which expressed high levels of VIM and were negative for CDH1, further corroborating the *in vitro* observation that *TMPRSS2/ERG* induces EMT. Staining for MKI67 (Ki-67), a known proliferation marker, revealed an extensive expression in the EP-AR *TMPRSS2/ERG* tumors (37% ±2 positive cells) compared to the EP-AR-derived nodules (8% ±2). This indicates that the EP-AR-derived nodules are less proliferative and may account for their latent nature.

**Figure 3:**
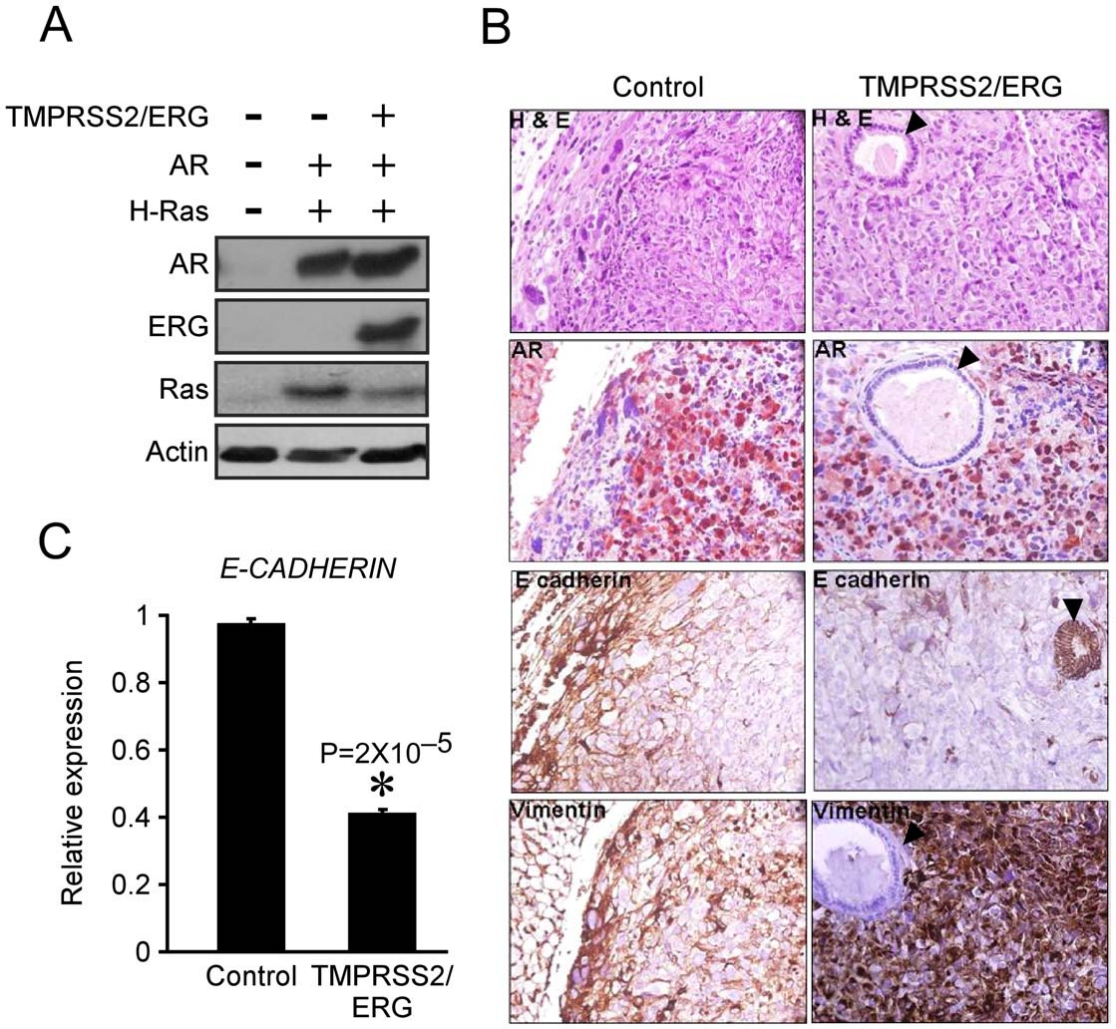
*TMPRSS2/ERG* modulates EMT markers in Ras-transformed prostate cells. (A) PrECs ectopically expressing hTERT as well as SV40 small and large T antigens were introduced with H-RasV12 and AR. The resulting line, LHSR (Control), was introduced with TMPRSS2/ERG to form the LHSR TMPRSS2/ERG line. A Western blot depicts the protein levels of the ectopically-expressed genes. (B) Cells were analyzed for CDH1 expression using QRT-PCR. The results are presented as mean ±SD of a triplicate from a representative experiment. (C) Cells were orthotopically implanted into mice prostate glands. Glands were removed one month after implantation, sectioned and stained with H&E or antibodies against AR, CDH1, and Vimentin. Black arrows denote mouse nodules with a negative staining for AR. (X400 Magnification).

The results described thus far suggest that *TMPRSS2/ERG* facilitates EMT and, consequently, the formation of more aggressive and proliferative tumors. Several studies demonstrated that compared to PIN lesions, *TMPRSS2/ERG* rearrangement frequency in localized invasive prostate cancers, is doubled [5], [28], [29], [30]. This observation implies that *TMPRSS2/ERG* requires additional modifications in order to be positively selected as the disease progresses. To test this hypothesis, we utilized a previously generated, Ras-transformed PrECs culture [22]. These cells harbor ectopically-expressed hTERT, the viral oncogenes SV40 small and large T antigens, oncogenic H-RasV12 and Androgen Receptor. An empty vector or a *TMPRSS2/ERG*-encoding vectors were introduced into these cells, to generate two distinct cell lines, LHSR and LHSR *TMPRSS2/ERG*, respectively (Figure 3A). In agreement with the results obtained with EP-AR cells, CDH1 was down-regulated in LHSR cells expressing *TMPRSS2/ERG* (Figure 3B). Next, cells were orthotopically injected into nude mice prostates, as well as sub-cutaneously. As expected, following merely 28 days, both cell lines gave rise to tumors with no significant differences in size (Tumor incidence is presented in Table S1). Accordingly, MKI67 staining revealed no differences between the cell lines in regards to proliferation rate (Data not shown). Interestingly, the *TMPRSS2/ERG*-expressing tumors demonstrated a marked up-regulation of VIM and a noticeable down-regulation of CDH1 compared to the control tumors (Figure 3C), further validating the facilitation of EMT by the *TMPRSS2/ERG* in an additional, and a more aggressive, *in vivo* model. Since the LHSR cell lines are highly aggressive, they are not suitable to study the effect of *TMPRSS2/ERG* on metastases formation, as the mice had to be sacrificed within a short period following the injections. Nevertheless, in one case, *TMPRSS2/ERG*-expressing tumor metastasized into the murine lung. As shown in Figure S1, this metastasis originated from the LHSR *TMPRSS2/ERG* primary tumor, as it stained positive with human-specific anti-AR antibody. It is tempting to speculate that EMT induced by the *TMPRSS2/ERG* granted cells with migratory and invasive capacities and eventually enabled them to home and proliferate at a distant site. Thus, given a highly transformed genetic background, *TMPRSS2/ERG*-induced EMT might facilitate invasion and metastasis.

### EP-AR and LHSR expressing TMPRSS2/ERG are not contaminated with cells of mesenchymal lineage

To exclude the possibility that the reported EMT stems from a cross contamination of mesenchymal cell cultures we performed Short Tandem Repeat (STR) based fingerprinting. STR loci are repetitive sequence elements, 3 to 7 base pairs in length, which are abundantly distributed throughout the human genome. PCR based STR analysis is increasingly being used as a means for human identification for forensic and linkage studies [31], [32], [33], [34]. We analyzed both EP and LHSR cultures based on allele assignment for each of the STR loci tested. EP-AR and EP-AR TMPRSS2/ERG were found to be identical with respect to the 16 STR loci (Table 1). LHSR and LHSR TMPRSS2/ERG were also found to be identical to each other, however not to EP-AR or EP-AR TMPRSS2/ERG. To corroborate this observation we also performed spectral Karyotying (SKY) analysis, in order to detect unique recurrent chromosomal features, specifically appearing in the two isogenic cell cultures. As shown in Figure S2, EP-AR and EP-AR TMPRSS2/ERG have additional material in chromosome 11, while the LHSR and LHSR TMPRSS2/ERG exhibit 3 specific chromosomal translocations, again indicating that each type of culture, stems from the same origin. These results suggest that the EMT reported herein is a genuinely induced by TMPRSS2/ERG rather than by cross-contamination.

**Table 1:** STR Alleles in EP and LHSR.

STR locus	Alleles: EP-AR and EP-AR TMPRSS2/ERG	Alleles: LHSR and LHSR TMPRSS2/ERG
D8S1179	14, 14	13, 16
D21S11	28, 30.2	31, 31.2
D7S820	8, 9	10, 11
CSF1PO	10, 11	12, 12
D3S1358	16, 16	15, 16
THO1	7, 9.3	9.3, 9.3
D13S317	11, 12	11, 13
D16S539	12, 13	10, 11
D2S1338	17, 20	17, 23
D19S433	11, 14	13, 13
vWA	16, 16	15, 16
TPOX	8, 8	8, 8
D18S51	17, 19	10, 13
Amelogenin	XY	XY
D5S818	12, 13	9, 10
FGA	22, 23	23, 24

### *TMPRSS2/ERG*-induced EMT is mediated by the *ZEB1*/*ZEB2* axis

**Figure 4:**
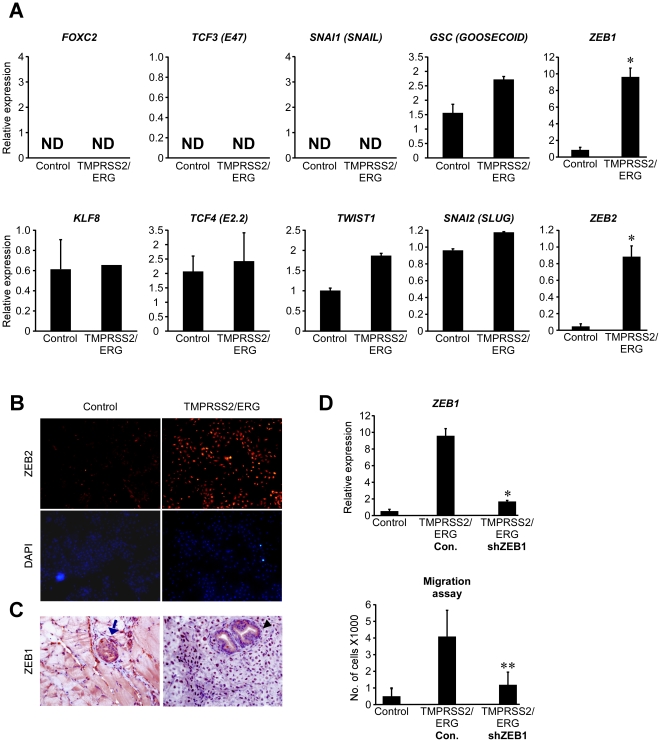
*TMPRSS2/ERG*-induced EMT is mediated by the *ZEB1*/*ZEB2* axis. (A) EMT-associated transcription factors expression. Cells were analyzed for the expression of the specified genes using QRT-PCR. The results are presented as mean ±SD of a triplicate from a representative experiment. ND  =  Not detected. * denotes a significant differential expression (P-Value<5×10^−4^). (B) Cells were plated on slides and stained with α-*ZEB1* antibody. Nuclei were visualized by DAPI. (C) Prostate glands were injected with EP-AR (Control) and EP-AR TMPRSS2/ERG as described, removed, sectioned and stained with an α-*ZEB1* antibody. The blue arrow denotes a human nodule. The black arrow denotes mouse nodules with a negative staining for AR. (X400 Magnification). (D) *ZEB1* was knocked-down and its mRNA levels were measured (upper panel). * denotes a significant differential expression (P-value = 8×10^−4^). Cells were subjected to migration assay as described (lower panel). ** denotes a significant differential expression (P-value = 4×10^−3^).

Numerous pathways are known to converge in *CDH1* repression during epithelial to mesenchymal transition [27]. Thus, we sought to measure the expression levels of several transcription factors which were reported to facilitate EMT by either direct or an indirect repression of *CDH1* [27]. The expression levels of *SNAI1 (Snail), SNAI2 (Slug), FOXC2, GSC (Goosecoid), TWIST1, TCF4 (E2.2), TCF3 (E47) and KLF8* were measured and found to be either low or equally expressed in both EP-AR and EP-AR *TMPRSS2/ERG* cell lines (Figure 4A). Remarkably, the expression of *ZEB1* and *ZEB2*, two known direct repressors of *CDH1* [27], were dramatically up-regulated in the *TMPRSS2/ERG*-expressing cells (Figure 4A). *ZEB1* induction by *TMPRSS2/ERG* was further validated at the protein level by immunostaining (Figure 4B). To test whether *ZEB1* has an effective role in promoting the EMT process in our model, its expression was stably knocked-down using short-hairpin RNA (shRNA) and migration assay was performed. As shown in Figure 4C, *ZEB1* levels declined dramatically following *ZEB1* knockdown, resulting in a significant attenuation of the migratory capacity of the *TMPRSS2/ERG*-expressing cells (Figure 4C, lower panel).

**Figure 5:**
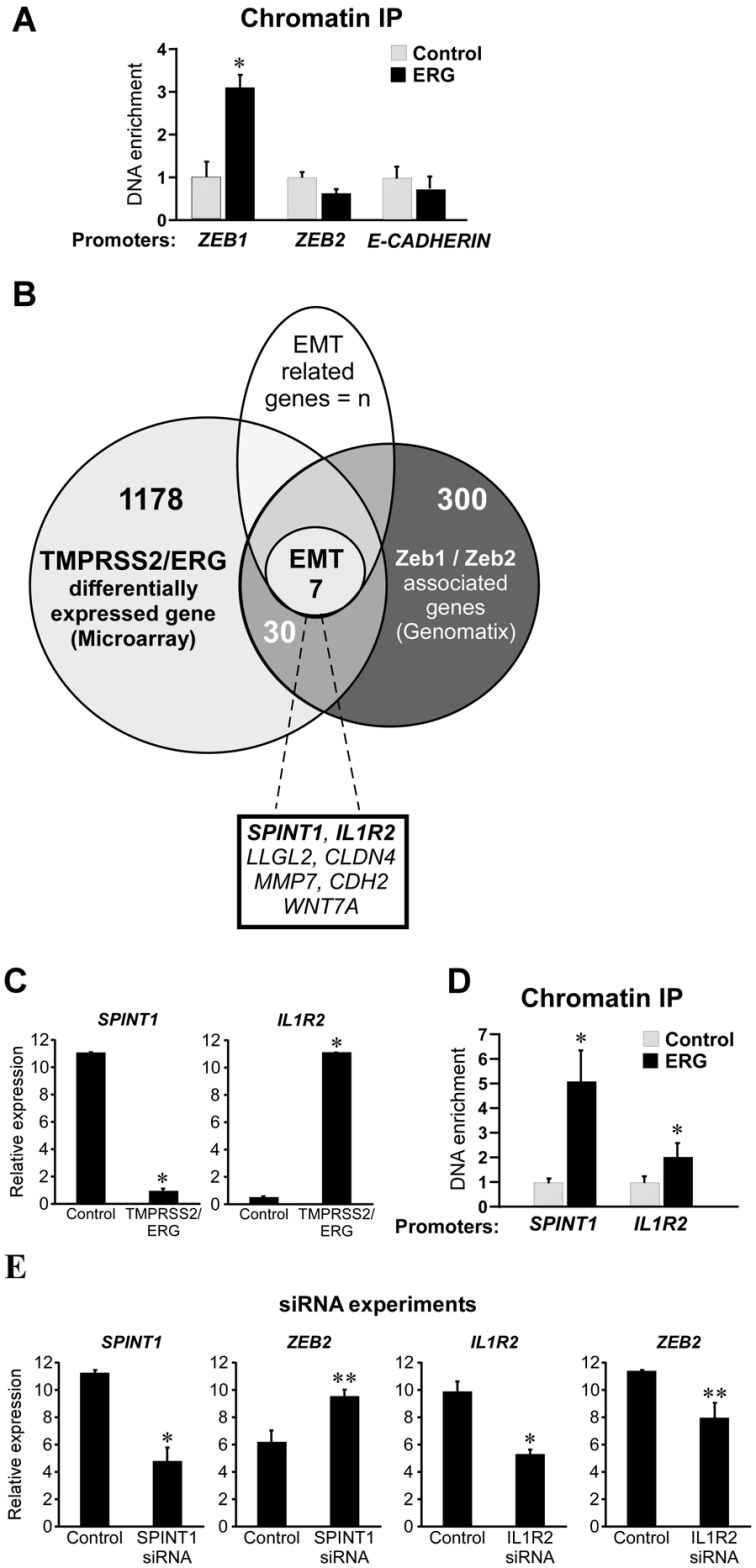
*TMPRSS2/ERG* induces an EMT transcription program. (A) Chromatin-IP assay was performed in EP-AR TMPRSS2/ERG cells using α-ERG antibody and IgG as a control. The results are presented as mean ±SD of a triplicate from a representative experiment. * denotes P-value<3×10^−2^. (B) The list of 1215 differentially expressed genes was intersected with a list of genes associated with *ZEB1* or *ZEB2*, which was obtained from the “Genomatix” software [47], resulting in 37 shared genes. The 37-genes list was filtered for genes associated with EMT according to the literature (n  =  unknown sample size), leaving the 7 genes that are listed in the box below. (C) Cells were analyzed for *SPINT1* and *IL1R2* expression using QRT-PCR. The results are presented as mean ±SD of a triplicate from a representative experiment. * denotes P-value<5×10^−4^. (D) Same set-up as (A) was used for *IL1R2* and *SPINT1* promoters (E) Left panels: EP-AR cells were transfected with siRNA targeting *SPINT1* and mRNA expression of the designated genes was measured by QRT-PCR. The results are presented as mean ±SD from two experiments utilizing two different siRNA oligonucleotides. * denotes P-value = 7×10^−4^, ** denotes P-value = 1×10^−3^. Right panels: the same experimental set-up was used with siRNA targeting *IL1R2* in EP-AR TMPRSS2/ERG. * denotes P-value = 2×10^−4^, ** denotes P-value = 1×10^−2^.

Both *ZEB1* and *ZEB2* promoter regions consist of putative *TMPRSS2/ERG* binding motifs (Figure S3), implying that *TMPRSS2/ERG* might directly bind their promoters and augment their expression. To test this hypothesis, we conducted a Chromatin Immuno-Precipitation (ChIP) assay using an α-ERG antibody. Interestingly, as depicted in figure 5A, *TMPRSS2/ERG* seems to directly bind *ZEB1* promoter, but not *ZEB2*. As a negative control we used a promoter region from *CDH1*, which is a part of the *ZEB1*/*ZEB2* axis, but does not harbor an ERG binding site. This result implies that *TMPRSS2/ERG* might indirectly induce *ZEB2* via the mediation of *ZEB2* up-stream effectors. To investigate this conjecture, and to better understand the mechanism by which *TMPRSS2/ERG* executes the EMT program at large; we undertook a genome-wide approach and conducted an expression micro-array-based comparison between EP-AR and EP-AR *TMPRSS2/ERG* cells. A total of 1215 annotated genes were differentially expressed between the two cell lines (813 up-regulated and 402 down-regulated), from which we retrieved the ones that were both associated with *ZEB1* or *ZEB2*, and implicated in EMT in the literature (For the detailed filtering method refer to legend of figure 5B). To verify the authenticity of this set of genes we also validated their microarray-derived differential expression patterns using QRT-PCR (Data not shown). As depicted in figure 5B, seven genes matched the filtering criteria. Two of them, *IL1R2* and *SPINT1* (Figure 5C, validated by QRT-PCR), were reported to encode upstream effectors of *ZEB2* expression; the former was shown to elevate *ZEB2* expression levels [35], while the latter attenuates its expression [36], suggesting that they might be the mediators of *TMPRSS2/ERG* dependent *ZEB2* elevation. Both *IL1R2* and *SPINT1* promoter regions consist of putative *TMPRSS2/ERG* binding motifs (Figure S2), and indeed *TMPRSS2/ERG* exhibited a significant binding to their promoters in a ChIP assay (Figure 5D). To further corroborate SPINT1 and IL1R2 effect on *ZEB2* expression in our system, we knocked-down their expression using small-interfering RNA (siRNA). As shown in figure 5E, *SPINT1* and *IL1R2* levels were effectively reduced upon siRNA transfection, resulting in *ZEB2* elevation and reduction, respectively. In sum, *TMPRSS2/ERG* seems to directly bind and trans-activate *ZEB1* while indirectly inducing *ZEB2* via trans-activation and trans-repression of its effectors, *SPINT1* and *IL1R2*.

## Discussion

In this study we provide substantial evidence to support the notion that *TMPRSS2/ERG* assumes an active role in epithelial to mesenchymal transition via the activation of the ZEB/*CDH1* pathway, both *in vitro* and *in vivo*. These findings shed light on the mechanism by which the *TMPRSS2/ERG* fusion exerts its oncogenic effect.

EMT and invasion capabilities were reported to be a consequence of *TMPRSS2/ERG* expression in several studies [10], [13], [15], [21]. Hence, it appears that EMT and invasion are general processes which are executed by *TMPRSS2/ERG* in prostate cancer, and which are achieved by diverse mechanisms. Consistently, WNT signaling components such as WNT7A, were evident in our model as well. EMT is induced by several signaling pathways, all funneled into the down-regulation of *CDH1*. These pathways are governed by approximately 10 major transcription factors, which repress *CDH1* expression in either a direct or an indirect fashion [27]. One such pathway is the SNAI1/2, which regulate *ZEB1*/2 expression and have been implicated in prostate cancer [37], [38], [39]. No differential expression of SNAI1 or SNAI2 was evident in our system. However, as previously reported [40], *ZEB* genes may promote EMT independently of Snail1/2 expression, which alludes to alternative, upstream activator of this particular pathway. In our system, *TMPRSS2/ERG* seems to meet the criteria required for this upstream activator.

AR plays a crucial role in prostate cancer progression and is known to regulate the *TMPRSS2* promoter, which constitutes the regulatory part of the *TMPRSS2/ERG* fusion. In addition, AR was recently shown to synergize with *TMPRSS2/ERG* to promote invasive adenocarcinoma development [20]. Accordingly, a strong cooperation between the two was evident in our system, as each gene did not activate and even slightly reduced *ZEB1/2* expression, while co-expression yielded a marked transcriptional induction of these genes (Data not shown). Similarly, while EP cells failed to grow *in vivo* following orthotopic implantation and EP-AR cells formed discrete nodules, EP-AR *TMPRSS2/ERG* cells gave rise to large malignant tumors, stressing the synergistic nature of their cooperation. Recent studies have linked AR to EMT in prostate cancer models through the activation of the SNAI axis [41], [42]. In our present study, AR alone was not sufficient to induce EMT, perhaps due to the Snail-depleted background. Thus, once again, it might be speculated that AR cooperates with *TMPRSS2/ERG* to invoke an alternative EMT pathway in the absence of Snail. The fact that the expression of both AR and *TMPRSS2/ERG* in our system is governed by artificial promoters, render it unsuitable to study AR induced *TMPRSS2/ERG* expression. However this system might represent the hormone refractory stage of advanced prostate cancer in which AR is over-activated. Our data implies that the cooperation between AR and *TMPRSS2/ERG* is not exclusively mediated through AR-dependent transcriptional regulation of ERG. Alternatively, other mechanisms, such as mediating components or physical interactions, may affect their cooperation in prostate cancer progression. Further studies should be focused on elucidating the exact mechanisms by which AR controls *TMPRSS2/ERG* expression and function.

Both SPINT1 and Il1R2 were previously implicated in cancer. Il1r2 was reported to be significantly elevated in the plasma of Hodgkin lymphomas' patients compared to healthy controls [43]. Moreover, forced expression of *IL1R2* in a uroepithelial cell line resulted in a morphological alteration, actin rearrangement and acquirement of a migratory capacity and *IL1R2* overexpression was associated with enhanced expression of *ZEB2* and reduced expression of *CDH1* [35]. Our work further corroborated *IL1R2* pro-oncogenic activity which is mediated by *TMPRSS2/ERG* direct trans-activation. Recently, in a study which compared tissues and cell lines representing different stages of Prostate cancer, Spint1 was highly expressed in normal tissues compared with Benign Prostatic Hyperplasia (BPH) and low-grade cancer, with a progressive loss in increasing tumor grade specimens [44]. Accordingly, *SPINT1* attenuation in prostate cancer cell lines, resulted in a more aggressive phenotype which included enhanced motility and invasiveness [45]. Finally, SPINT1 knockdown in the pancreatic cancer cell line SUIT-2, induced EMT and invasion which were accompanied by *ZEB2* elevation and *CDH1* reduction [36]. Collectively these data suggest that SPINT1 acts as a tumor suppressor in Prostate cancer. We were able to show that SPINT1 partially exerts its effect by reducing the levels of *ZEB2* and therefore it is repressed by *TMPRSS2/ERG*.

**Figure 6:**
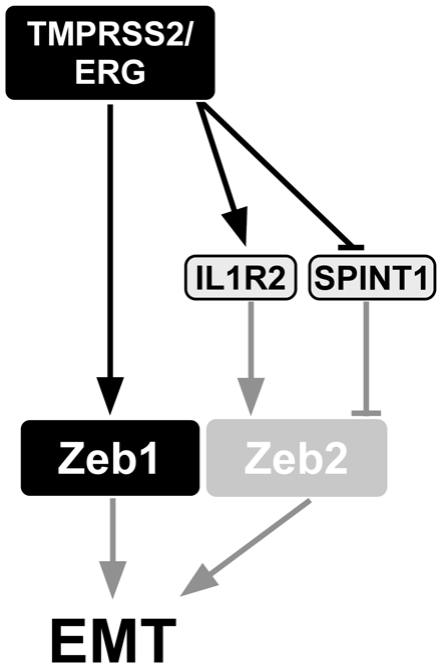
A schematic model describing the proposed mechanism by which *TMPRSS2/ERG* induces EMT. Black lines represent novel data; arrowheads represent activation; bar-headed lines represent repression; grey lines represent literature-based data.

Recently, reports which focus on the cooperation of *TMPRSS2/ERG* with different partners in the cancerous process are emerging [16], [19], [20]. Our findings (Depicted in figure 6) extend our knowledge as to the identity of these partners and their mechanisms of action in promoting prostate cancer.

## Materials and Methods

### Cell lines propagation

Human PrECs were obtained from BioWhittaker (Rockland, ME) and propagated in Prostate Epithelial Growth Medium (Lonza, Walkersville, MD) as previously described [22]. Cells were maintained in a humidified incubator at 37°C and 5% CO2.

### Retroviral infections

Retroviral infections were performed serially and polyclonal-infected populations were drug-selected after each infection. Amphotropic retroviruses were produced by transfection of 293T cells with amphotropic packaging plasmid pCL-10A1, and a retroviral vector encoding the gene of interest. Culture supernatants containing retrovirus were collected 48 hours post-transfection.

### *TMPRSS2/ERG* plasmid

TMPRSS2 (exon-1) and ERG (exons 4-9) fusion-encoding pBabe-Hygro plasmid, was kindly provided by Dr. Jan Trapman (Erasmus University Medical Center, Rotterdam, the Netherlands).

### Orthotopic implantation of tumor cells

The protocol for *in vivo* experiments was approved by the Sheba Medical Center Institutional Animal Care and Use Committee (Permit No. 468–2008). Mice were anesthetized prior to injections and sacrificed when tumor size reached 1 cm^3^. All efforts were made to minimize animals' suffering. Immunodeficient mice (Harlan Laboratories, Israel) were anesthetized with a mixture of 100 mg/kg ketamine and 10 mg/kg Xylazin 2∶1 (Ketaset). A lower midline incision was made; and 1×10^6^ cells (in 100 µl PrEGM:matrigel (BD Bioscience) 1∶1 mix), were implanted into the ventral prostate lobes using a 30-gauge needle and a 0.1 mL syringe. Testosterone pellets (Innovative Research of America, Sarasota, FL) were implanted under the skin. Two Subcutaneous injections were placed for each mouse as well. Mice were sacrificed at 28 or 68 days, as indicated, after the intraprostatic implantation of tumor cells. A table summarizing tumor incidence is presented as Table S1.

### Migration and invasion assays

Cells were plated at a cell density of 1.5×10^5^ per well in 8 µm transwells (Co-Star) in triplicates and incubated for 24 hours. Then, cells that were attached to the outer part of the wells were removed by incubation with trypsin for 30 minutes and counted. For invasion assays, the transwells were coated with matrigel diluted in cell media 1∶5. Cells were seeded in the presence of basal medium and migrated towards 10% FCS.

### Western blot analysis

Total cell lysates were fractionated by SDS-gel electrophoresis. Proteins were transferred to nitrocellulose membranes, and immunoblotted with the indicated antibodies. Rabbit α-p53 (produced in Rotter's lab); human specific α-Androgen Receptor (α-hAR N-20, Santa-Cruz Biotechnology); α-ERG (SC-354, Santa-Cruz Biotechnology); α–H-ras (C-20, Santa-Cruz Biotechnology); α-hTERT (H-231, Santa-Cruz Biotechnology); α-cyclin D2 (C-17, Santa-Cruz Biotechnology); α-actin (I-19, Santa-Cruz Biotechnology). Bands were detected by horseradish peroxidase–conjugated secondary antibodies and enhanced SuperSignal west pico chemiluminescent substrate (Thermo-scientific).

### Quantitative Real-Time PCR (QRT-PCR)

RNA was isolated using TRIzol (Invitrogen) according to the manufacturer's instructions. cDNA was generated from a 2- µg aliquot of the RNA using MMLV reverse transcriptase, amplification grade DNase I, random hexamer primers, RNaseOUT, and dinucleotide triphosphates (all from Invitrogen), according to the manufacturer's instructions. QRT-PCR was performed using SYBR-Green Master Mix (Applied-Biosystems, CA, USA) on a 7500 Real-Time PCR system (Applied-Biosystems, CA, USA). Gene expression was normalized to GAPDH. Primers sequences are listed in Table S2.

### Immunohistochemistry

Xenografts were fixed in formalin, embedded in paraffin and sectioned at 4 µm. The slides were incubated at 60°C for one hour. After sections were dewaxed and rehydrated, a CC1 Standard Benchmark XT pretreatment for antigen retrieval was selected (Ventana-Medical Systems). α-Vimentin (NCL-L-VIM-572, Leica Novocastra) was diluted 1∶100. α-Ki67 antibody (MU297-UC, Biogenex), was diluted 1∶50. α-*CDH1* antibody (18-0223, Zymed) was diluted 1∶25. Antibodies were incubated for 40 minutes at 37°C. Detection was performed with iView detection kit (Ventana-Medical Systems) and counterstained with hematoxylin (Ventana-Medical Systems). Then, slides were dehydrated in 70% ethanol, 95% ethanol and 100% ethanol for 10 seconds each. Before coverslipping, the sections were cleared in xylene for 10 seconds and mounted with Entellan.

For AR staining, antigen retrieval was performed using a pressure cooker (Milestone, Microwave-Laboratory Systems) at 120°C for 5 minutes in citrate buffer pH 6, cooled for 10 minutes, and rinsed with TBS buffer. Subsequently, an endogenous peroxidase block was performed for 10 minutes in 3% H_2_O_2_/PBS. After TBS rinsing, sections were blocked with 10% goat serum for 30 minutes and incubated with the α-hAR primary antibody (N-20, Santa-Cruz Biotechnology, 1∶50) overnight at 4°C. Detection was performed with the Histostain SP Broad Spectrum kit (Zymed Laboratories, Invitrogen, U.S.A.). Briefly, sections were incubated with a biotinylated secondary antibody and subsequently, after TBS rinse, with HRP-streptavidin, for 30 minutes at room temperature. The antibody was visualized with the substrate-chromogen AEC, counterstained with hematoxylin and coverslipped with an aqueous mounting fluid (glycergel).

### SKY analysis

Described in detail in [46].

### Expression micro-arrays

Experiments were performed using Affymetrix GeneChip Human Gene 1.0 ST Arrays according to manufacturer's recommendations. Briefly, 100–600 ng of total RNA was used to generate first-strand cDNA using random hexamers primer. After second-strand synthesis, *in vitro* transcription was performed. The resulting cRNA was then used for a second cycle of first-strand cDNA with UTP resulting in single-stranded DNA which was used for fragmentation and terminal labeling. cDNA generated from each sample was processed as per manufacturer's recommendation using an Affymetrix GeneChip Instrument System manual (https://www.affymetrix.com/support/downloads/manuals/wt_sensetarget_label_manual.pdf).

### Mircro-array data analysis

EP-AR and EP-AR *TMPRSS2/ERG* expression profiles were analyzed on duplicate arrays. Gene level RMA sketch algorithm (Affymetrix Expression Console and Partek Genomics Suite 6.2) was used for crude data generation. A t-test with an uncorrected P-value was used to identify significantly differentially expressed genes (P-value<0.05), with a threshold of at least two-fold change. This analysis yielded a set of 1215 differentially expressed genes.

### Chromatin immunoprecipitation

Cells underwent cross-linking (1%formaldehyde, room temperature, 10 minutes) followed by quenching (glycine 0.125 M). Cells were rinsed with cold PBS, incubated with 20% trypsin (Gibco), washed with PBS, scraped and centrifuged. Cells were lyzed (5 mM PIPES pH 8.0, 85 mM KCl, 0.5%NP40, 1%protease inhibitors) on ice for 20 minutes. Nuclei were collected by centrifugation (4,000 rpm), resuspended in nuclear lysis buffer (50 mM Tris–Cl, pH 8.1, 10 mM EDTA, 1%SDS, 1%protease inhibitors) and incubated on ice for 10 min. Samples were sonicated to an average DNA fragment length of 500 bp and then centrifuged (20,000g). The chromatin solution was pre-cleared by adding protein A beads (2 hours, 4°C) (Santa Cruz Biotechnology). Immunoprecipitation of chromatin was done for 12 hours, in 4°C, using 1 µl antibody (α-ERG SC-354, Santa-Cruz Biotechnology and IgG I-2511, Sigma), followed by incubation with 30 µl protein A beads (2 hours). Immunoprecipitates were consecutively washed with dilution buffer (100 mM Tris–Cl, pH 9.0, 500 mM LiCl, 1%NP-40, 1%Deoxycholic acid, 1% protease inhibitors), TSE150, TSE500 and TE pH = 8. Samples were treated with 10 µg RNase A (30 minutes), followed by 30 µg of proteinase K treatment (2 hours, 50°C) and incubation at 65°C overnight. DNA samples were extracted using QIAquick PCR Purification Kit (Qiagen). QRT-PCR was performed as described above with each sample containing 2 µl of immunoprecipitated DNA. Primers sequences are listed in Table S2.

### Short tandem repeat (STR) based fingerprinting

DNA was amplified by PCR using the reagents supplied in the AmpF*l*STR^R^ Identifiler Plus (Applied Biosystems, Foster City, 94404 Ca., USA) for the following STR loci: D8S1179, D21S11, D7S820, CSF1PO D3S1358, TH01, D13S317, D16S539, D2S1338, D19S433, vWA TPOX, D18S51, Amelogenin, D5S818, and FGA. The products were separated on an Applied Biosystems, 3130 genetic analyzer and analyzed using the software supplied by the manufacturer.

## Supporting Information

Figure S1 **The formation of tumor metastasis.** LHSR T/ERG tumor metastasized into the murine lung and stained for AR (right hand side) compared to the normal lung of the LHSR mouse (left hand side) (X400 Magnification). Arrow in the LHSR T/ERG panel indicates lung metastasis.
(TIF)

Figure S2 **EP-AR and LHSR exhibit identical chromosomal characteristics as their TMPRSS2/ERG expressing counterparts.** The designated cell cultures were subjected to SKY analysis. (A) Most recurrent features are shown in a table. (B) Representative images of the chromosomal features, recurrent features are circled in white.
(TIF)

Figure S3 **ERG binding sites in various promoters.** (A) The ETS transcription family binding site sequence. (B) ∼2000 base pairs up stream to the translation start site of the gene of interest were analyzed using ‘MatInspector’ Algorithm by ‘Genomatix’. ERG putative binding sites which passed a threshold of >0.96 matrix similarity and a core similarity of 1 are depicted in the table. In the sequence columns, capital letters represent core sequence.
(TIF)

Table S1 **Tumor incidence summary.**
(TIF)

Table S2 **Primers list.**
(DOC)
