# Supplementary figures and images for: TMPRSS2/ERG Promotes Epithelial to Mesenchymal Transition through the ZEB1/ZEB2 Axis in a Prostate Cancer Model
Source: PLoS One. 2011 Jul 1;6(7):e21650. doi: 10.1371/journal.pone.0021650 (PMC3128608; doi:10.1371/journal.pone.0021650)

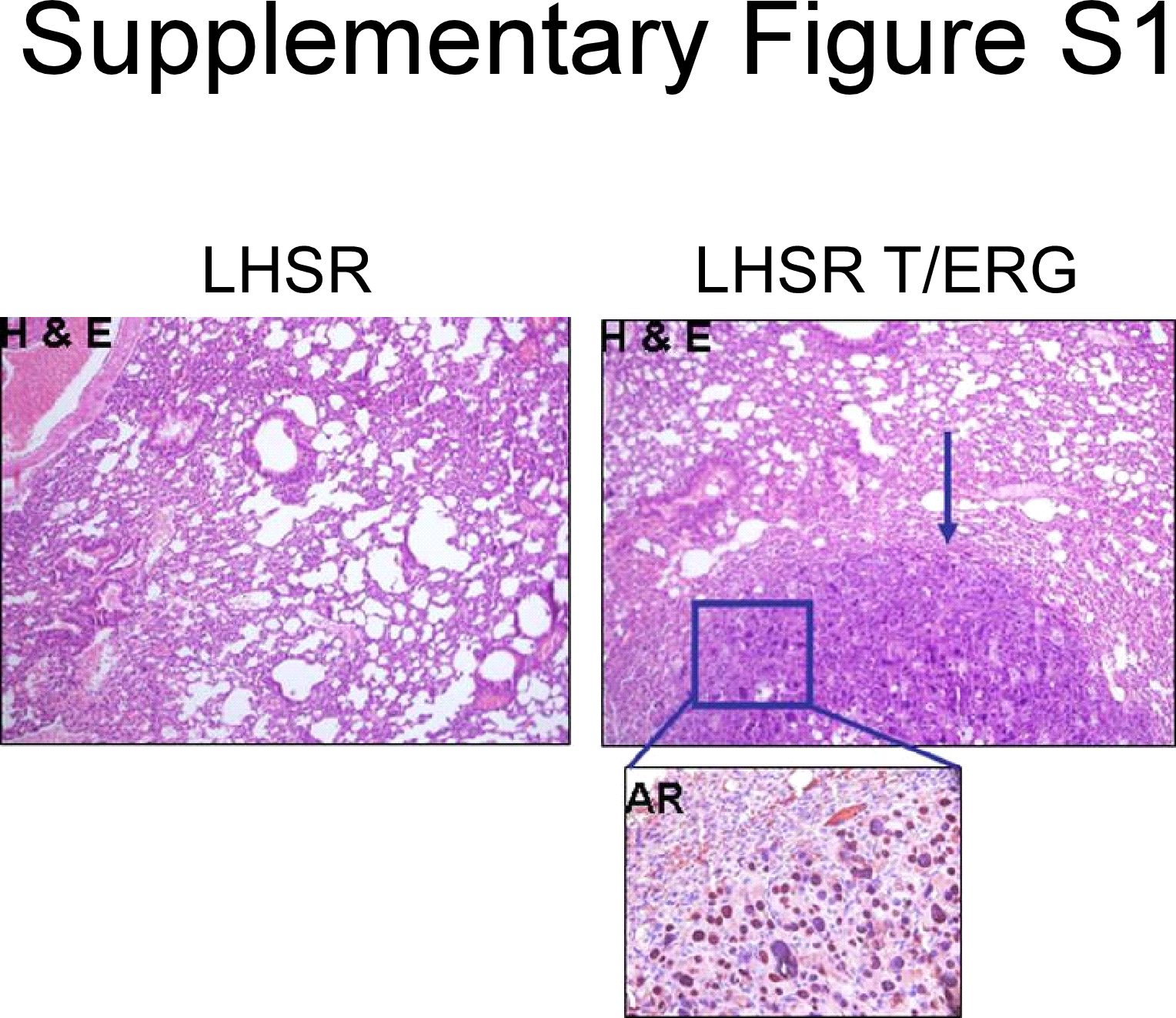

Supplement: Figure S1 — The formation of tumor metastasis. LHSR T/ERG tumor metastasized into the murine lung and stained for AR (right hand side) compared to the normal lung of the LHSR mouse (left hand side) (X400 Magnification). Arrow in the LHSR T/ERG panel indicates lung metastasis. (TIF) [file pone.0021650.s001.tif]

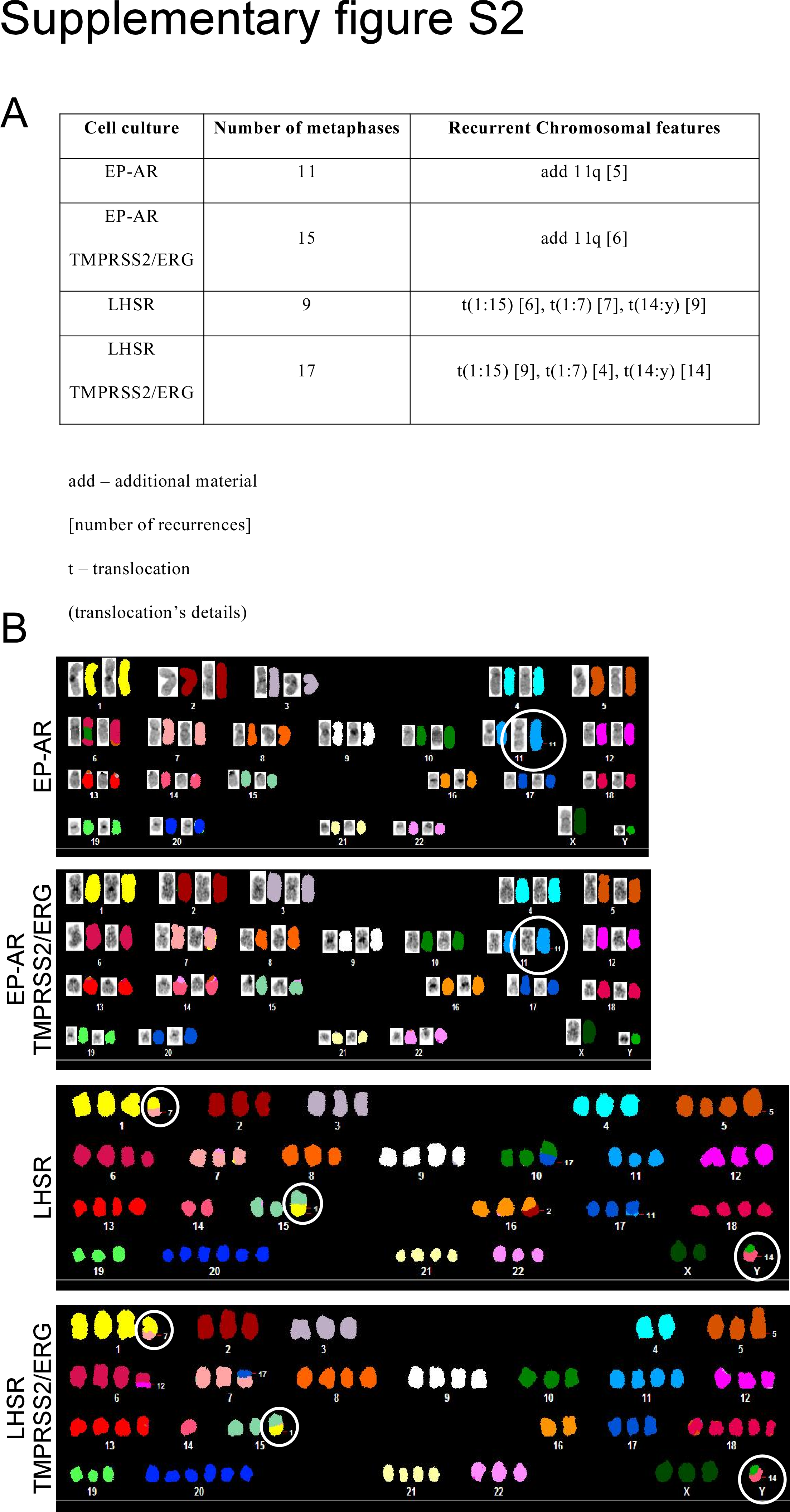

Supplement: Figure S2 — EP-AR and LHSR exhibit identical chromosomal characteristics as their TMPRSS2/ERG expressing counterparts. The designated cell cultures were subjected to SKY analysis. (A) Most recurrent features are shown in a table. (B) Representative images of the chromosomal features, recurrent features are circled in white. (TIF) [file pone.0021650.s002.tif]

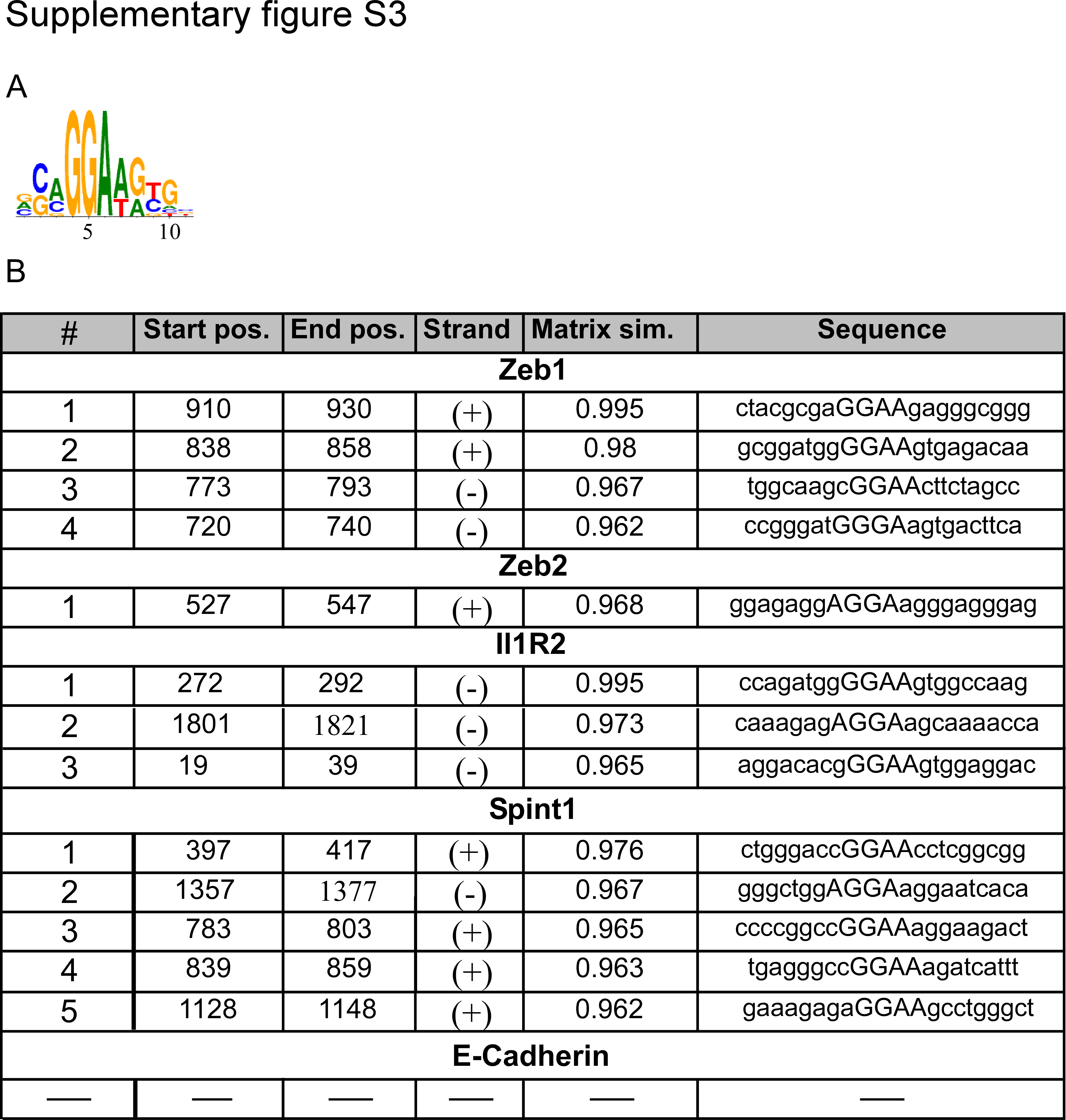

Supplement: Figure S3 — ERG binding sites in various promoters. (A) The ETS transcription family binding site sequence. (B) ∼2000 base pairs up stream to the translation start site of the gene of interest were analyzed using ‘MatInspector’ Algorithm by ‘Genomatix’. ERG putative binding sites which passed a threshold of >0.96 matrix similarity and a core similarity of 1 are depicted in the table. In the sequence columns, capital letters represent core sequence. (TIF) [file pone.0021650.s003.tif]

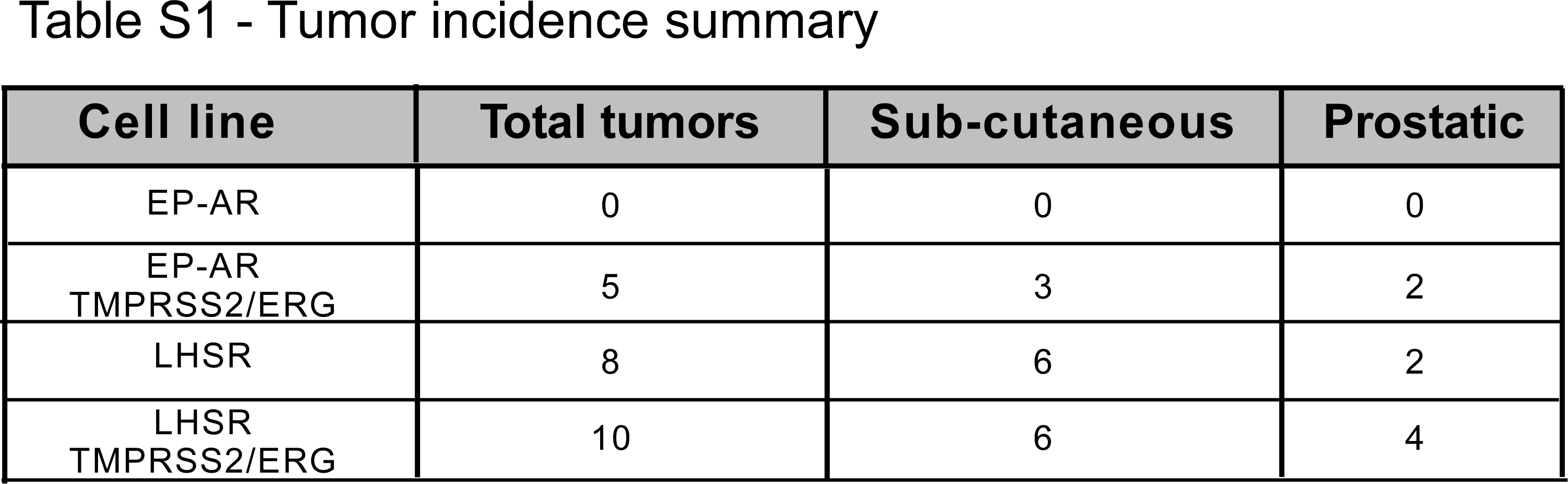

Supplement: Table S1 — Tumor incidence summary. (TIF) [file pone.0021650.s004.tif]
