# Supplementary material for: TMPRSS2/ERG Promotes Epithelial to Mesenchymal Transition through the ZEB1/ZEB2 Axis in a Prostate Cancer Model
Source: PLoS One. 2011 Jul 1;6(7):e21650. doi: 10.1371/journal.pone.0021650 (PMC3128608; doi:10.1371/journal.pone.0021650)
Supplement: Table S2 — Primers list. (DOC) [file pone.0021650.s005.doc]

**Table S2**

QRT-PCR

| **Gene symbol** | **Sequence** |
| --- | --- |
| CDH1 forward | GCATTGCCACATACACTCTCTTCT |
| CDH1 reverse | TGCATTCCCGTTGGATGAC |
| CDH2 forward | TGACCAGCCTCCAACTGGTAT |
| CDH2 reverse | GCATGTGCCCTCAAATGAAA |
| CLDN4 forward | TCTGCGAACGTTAAGTCCGTC |
| CLDN4 reverse | CATTACCTGTAGCCCCATGGA |
| FoxC2 forward | GCCCAGCAGCAAACTTTCC |
| FoxC2 reverse | CCGGTGGGAGTTGAACATCT |
| GSC forward | AGCAGCTCGAAGCTCTCGAG |
| GSC reverse | CACGTCCGGGTACTTGGTCT |
| IL1R2 forward | CTACGCACCACAGTCAAGGAAG |
| IL1R2 reverse | CGTCTGTGCATCCATATTCCC |
| KLF8 forward | TCAGAAGGTGGCTCAATGCA |
| KLF8 forward | TCAGAAGGTGGCTCAATGCA |
| KLF8 reverse | CCGAACAGAAGCAGTGACCTG |
| KLF8 reverse | CCGAACAGAAGCAGTGACCTG |
| MMP7 forward | ATCCCCCTGCATTTCAGGAA |
| MMP7 reverse | TTCCTGGCCCATCAAATGG |
| SNAI1 forward | TGGTTCTTCTGCGCTACTGCT |
| SNAI1 reverse | GTGGTTCTTCTGCGCTACTGCT |
| SNAI2 forward | GAAGATGCATATTCGGACCCA |
| SNAI2 reverse | CCGCAGATCTTGCAAACACA |
| SPINT1 forward | AGACTACTGCCTCGCATCCAA |
| SPINT1 reverse | CAAGCAGCCTCCATAAACGAA |
| TCF3 forward | AGCTCCTCCTTTGACCCCAG |
| TCF3 reverse | ACTCAGTGAAGTGGGTGCCC |
| TCF4 forward | TGCGATGTTTTCACCTCCTG |
| TCF4 reverse | TGCCAAAGAAGTTGGTCCATT |
| WNT7A forward | TGTGGCTGCGACAAAGAGAA |
| WNT7A reverse | TCCACAAAGACCTTGGCGA |
| Zeb1 forward | AGGTGTAAGCGCAGAAAGCAG |
| Zeb1 reverse | TCTTGCCCTTCCTTTCTGTCA |
| Zeb2 forward | TTCGCTTCCACCTTTTTCTCC |
| Zeb2 reverse | AGGCTCGATCTGCGAAGTCTT |

**Chromatin IP – ERG binding sites**

| **Gene symbol** | **Sequence** |
| --- | --- |
| CDH1 forward | TGGCCAACATGGTGAAACCC |
| CDH1 reverse | GAGTGCAGTGGCACGATCTC |
| IL1R2 forward | TTAACTCCAGCATGGCCAAG |
| IL1R2 reverse | CGCTCTGTGCTCAACTAAAC |
| SPINT1 forward | TACCTTCCCGCCACGAAATG |
| SPINT1 reverse | GGCCTCCAGTCTTCCTTTCC |
| Zeb1 forward | GCTCTGAGTCCTGCCACCTA |
| Zeb1 reverse | CGGACTCCCTTCCCTTCC |
| Zeb2 forward | AATGATGCCAAAGGCTTGAC |
| Zeb2 reverse | AAGTCTCCGCAAACGCTTTA |
